# Pulmonary hypertension: Past, present and future

**DOI:** 10.4103/1817-1737.37832

**Published:** 2008

**Authors:** Robyn J. Barst

**Affiliations:** *Pulmonary Hypertension Center, Columbia University and Cornell Medical Center, New York, NY, USA*

Pulmonary arterial hypertension (PAH) is a progressive disease characterized by an elevation of pulmonary artery pressure and pulmonary vascular resistance, leading to right ventricular failure and death. Idiopathic PAH (IPAH; formerly termed primary pulmonary hypertension) occurs in the absence of known causes. Estimates of the incidence of IPAH and familial PAH (FPAH) range from 1-2 cases per million people in the general population, with at least 6% of these patients having FPAH. Although the incidence of PAH in patients with other illnesses is not known with certainty, from various reports it appears that 2-4% of patients with portal hypertension and 0.1-0.6% of HIV patients have PAH. The incidence of PAH that occurs in patients with connective tissue disease is extremely variable; prevalence ranges from 2 to 35% in patients with the scleroderma spectrum of disease and may reach as high as 50% of patients with limited scleroderma. PAH has also been reported to occur in 10-45% of patients with mixed connective tissue disease and in 1-14% of cases with systemic lupus erythematosus. The incidence of PAH associated with anorexigens is cyclical in nature and varies depending on the availability of specific appetite suppressants. The link was first identified in the 1960s when an epidemic of PAH occurred in Switzerland, Austria and Germany that was linked to the anorexigen aminorex fumarate. Use of the anorexigens fenfluramine and dexfenfluramine have also been linked with an increased risk for PAH.

Prior to the development of disease-specific targeted PAH therapies, the median survival for subjects diagnosed with IPAH was approximately 2.8 years. However, 2.8 years likely underestimates current survival as the course of the disease has been favorably altered by therapeutic advances since that report from the 1980s. Prognosis is also dependent on the underlying etiology of the disease. The prognosis for patients with PAH associated with connective tissue disease appears to be worse than for those with IPAH. Estimates for 2-year survival in scleroderma patients with associated PAH are 40% compared with 48% for 3-year survival in patients with IPAH. Survival in patients with HIV-associated PAH is similar to patients with IPAH. With current HIV therapies, most of the deaths in patients with HIV and associated PAH are now attributed to PAH.

**Figure 1:**
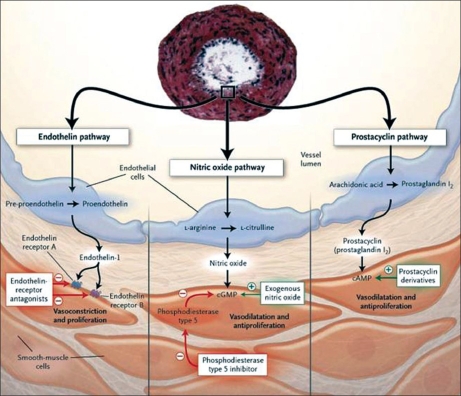
Targeted medical therapy for pulmonary arterial hypertension based on the prostacyclin pathway, the nitric oxide pathway and the endothelin pathway. Reprinted with permission from Humbert *et al.*, *N Engl J Med* 2004

**Figure 2:**
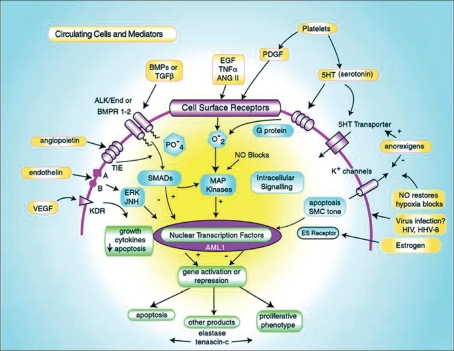
Some cellular processes implicated in the pathogenesis of PAH. Extracellular mediators and cells (platelets) are highlighted in yellow, cell surface receptors and ion channels in purple, intracellular signaling in blue and nuclear responses in green. See text for detailed descriptions of pathogenic mechanisms and interactions among the many pathways that span the extracellular, membrane, cytosolic and nuclear domains. VEGF indicates vascular endothelial growth factor; its receptor is KDR. Intracellular transduction of this pathway is poorly understood. Endothelin is vasoactive and a mitogen, acting through Ca^2 +^ channels and ERK/Jun kinases. Tyrosine kinase is the angiopoietin receptor, a system found to be upregulated in pulmonary vascular disease.^56^ Alk 1 and BMPR1-2 are receptors of the TGF-β superfamily, and BMP indicates bone morphogenetic protein. Alk 1 mutations cause hereditary hemorrhagic telangiectasia and some cases of Idiopathic Pulmonary Arterial Hypertension. Epidermal growth factor (EGF), tumour necrosis factor (TNF)-, angiotensin II (ANGII) and platelet-derived growth factor (PDGF) are all proliferative stimuli that act through tyrosine kinase receptors and are partially transduced by intracellular oxidant species. In the intracellular domain, SMADs are regulatory proteins that activate nuclear transcription factors and interact with MAP kinases. AML 1 is a nuclear transcription factor of potential importance. Elastase, downstream of AML 1, has been implicated in vascular disease in experimental animals. Viral proteins are found in vascular lesions in the lungs of patients with PAH, raising the possibility that they participate in its pathogenesis. Reprinted with permission from

Although Ernst von Romberg, a German physician, described an autopsy in 1891 as ‘pulmonary vascular sclerosis,’ it is only since 1995 with the introduction of intravenous epoprostenol that disease-specific targeted medical therapies for PAH have become available. In addition, significant advances in the treatment of PAH have occurred during the past decade, with six medical therapies now having received regulatory approval worldwide targeting the prostacyclin pathway, the nitric oxide pathway and the endothelin pathway [Figure 1]. Furthermore, ongoing clinical trials are evaluating novel therapeutic approaches based on scientific insights gleaned over the past decade in the pathobiology of PAH [Figure 2].

**Figure 3:**
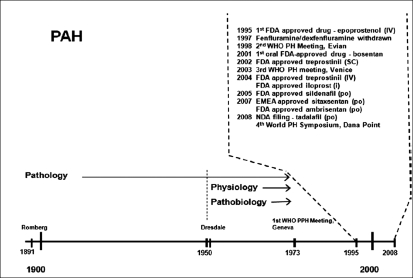
Pulmonary arterial hypertension: a historical perspective

From a therapeutic standpoint, why had it taken from 1891 until 1995 to develop a safe and efficacious therapeutic modality for the treatment of PAH? [Figure 3] Although several reports of young women dying of right heart failure without a diagnosis were published in 1940, it was not until pulmonary artery pressures could be recorded directly with the introduction of right heart catheterization that the physiology of the pulmonary circulation could be studied. In 1951, Dresdale tested the acute effects of tolzoline in a young woman with IPAH; the tolzoline caused a sudden decrease in pulmonary artery pressure and pulmonary vascular resistance without significant systemic effects. Unfortunately, no drugs were available at that time for chronic treatment. However, despite this, there remained little interest in PAH until the epidemic of the aminorex-induced PAH became apparent in the late 1960s. Prompted by the aminorex-induced PAH epidemic in 1973, the World Health Organization (WHO) held its first meeting in Geneva to assess what was known about IPAH and what remained unknown. In 1981, the National Heart, Lung and Blood Institute of the National Institutes of Health supported a national registry of patients with IPAH, which resulted in several reports over the next decade describing clinical features of IPAH and its natural history. Interestingly, despite the fact that IPAH was an orphan disease, significant interest from the scientific community rapidly ensued. Advances in the understanding of the mechanisms involved in the pathobiology of IPAH and PAH associated with other conditions have focused on molecular biology, developmental biology and genetics. Together with epidemiological and natural history studies, collaborative efforts between the scientific community and industry have led to a surge in clinical trials over the past decade: since the approval of intravenous epoprostenol for the treatment of IPAH in 1995, the prostacyclin analogue treprostinil has been approved for continuous subcutaneous infusion in 2002 and for continuous intravenous infusion in 2004. In addition, the prostacyclin analogue iloprost was approved in 2004 via inhalation. In 2001, bosentan, an endothelin ET _A_/ET_B_ receptor antagonist, was the first oral therapy approved for the treatment of PAH; and sildenafil citrate, an oral phosphodiesterase type 5 inhibitor, was approved in 2005. In 2007, the oral ET_A_ selective ERA ambrisentan was approved, and the oral ET_A_ selective ERA sitaxsentan was approved in the EU.

**Figure 4:**
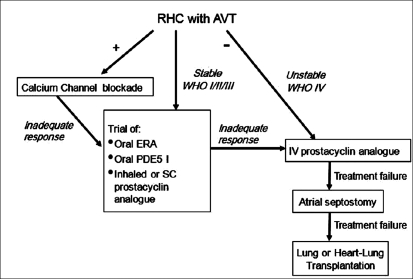
Current consensus evidence-based guidelines for the treatment of pulmonary arterial hypertension

Prompted by the scientific insights from the 1990s, in 1998 the second WHO meeting was held on the 25^th^ anniversary of the original meeting; and with the dramatic advances over the next 5 years, the 3^rd^ WHO Symposium on PAH was held in 2003 and the 4^th^ World Symposium on PAH in 2008. Based on the clinical trials to date, current consensus evidence-based guidelines for the treatment of PAH are shown in [Figure 4]. What have we been able to achieve? The disease-specific PAH therapies, currently available in conjunction with anticoagulant, diuretic, digitalis and oxygen therapy, have improved exercise capacity, functional capacity, time to clinical worsening, hemodynamic parameters, overall quality of life and survival. However, PAH remains a devastating, life-threatening disorder. In more than 50% of patients, exercise capacity remains significantly limited, approximately 50% of patients remain WHO functional class III or IV, PAH patients continue to have frequent hospitalizations for PAH, right heart function remains significantly impaired in most patients, quality of life is suboptimal and despite an increase in survival for functional class III and IV patients with IPAH from a predicted survival of 33% (based on the NIH Registry) to 63% with our current therapeutic modalities, the outlook is far from ideal; we need to continue to aggressively pursue furthering our understanding of PAH if we ever hope to give these patients a near-normal life.

We believe that future developments in vascular biology will improve our understanding of the pathobiology of PAH and provide rationale and ‘proof of concept’ for more disease-specific targeted therapies. With the advent of genomic technologies and methods, the necessary tools are now becoming available to begin pinpointing the genes that contribute to disease susceptibility and progression. Candidate gene discovery, that is, gene analysis using microarrays, can identify genes that may provide valuable insight into disease biology and may represent an initial step towards the identification of genetic polymorphisms that may help predict efficacy or lack thereof, with various disease-specific targeted PAH therapeutic modalities. By identifying the genes and gene variants that determine individual disease susceptibility, we might be able to one day identify patients in pre-clinical stages of disease as well as allow for individualized therapies that are most efficacious and least likely to cause side effects. Furthermore, although right ventricular function appears to be the most significant prognostic parameter in PAH, comparatively little attention has been devoted to how right ventricular function and dysfunction can be detected and measured, what specific molecular and cellular mechanisms contribute to maintenance or failure of right ventricular function, how right ventricular dysfunction evolves structurally and functionally or what interventions might best preserve right ventricular function. In addition, right ventricular-left ventricular interaction and right ventricular-pulmonary arterial coupling have largely been overlooked as potential targets for investigation and therapy. Whether cell-based or gene therapy, in addition to new drugs or new combinations of existing drugs targeting right heart failure, in conjunction with PAH-specific vasodilator and antiproliferative drugs will improve outcomes in PAH will require further study. Ultimately, as these novel therapeutic options are developed, individualized treatment regimens will evolve. However, many questions remain regarding the treatment of patients with PAH, e.g., identification of patient populations who will most benefit from a specific therapy, determining when treatment should be initiated and establishing optimal drug sequencing and combinations. We hope that by further increasing our understanding of the pathobiology of PAH, we will one day be able to prevent and cure this disease.

However, in the interim, it is imperative that we base our treatment regimens on evidence-based studies. As stated by Hippocrates in Precepts (∼440 B.C.E.), “In Medicine one must pay attention not to plausible theorizing but to experience and reason together… I agree that theorizing is to be approved, provided that it is based on facts and systematically makes its deductions from what is observed… But conclusions drawn from unaided reason can hardly be serviceable; only those drawn from observed fact.”

